# Impact of traumatic brain injury on risk for schizophrenia and bipolar disorder

**DOI:** 10.1016/j.psychres.2024.115990

**Published:** 2024-06-02

**Authors:** Kai-Yuan Cheng, Natassia Robinson, Alexander Ploner, Ralf Kuja-Halkola, Yasmina Molero, Paul Lichtenstein, Sarah E. Bergen

**Affiliations:** a Department of Medical Epidemiology and Biostatistics, Karolinska Institutet, Nobels väg 12A, SE-17177 Stockholm, Sweden; b Centre for Psychiatric Research, Karolinska Institutet, Norra Stationsgatan 69, SE-113 64 Stockholm, Sweden

**Keywords:** Head injuries, Psychotic disorders, Family design, Sibling comparison

## Abstract

The impact of traumatic brain injury (TBI) on subsequent risk of schizophrenia (SCZ) or bipolar disorder (BD) remains contested. Possible genetic and environmental confounding effects have also been understudied. Therefore, we aim to investigate the impact of TBI on the risk of SCZ and BD and whether the effect varies by injury severity, age at injury, and sex. We identified 4,184 SCZ and 18,681 BD cases born between 1973 and 1998 in the Swedish National Registers. Case-control samples matched (1:5) on birth year, sex, and birthplace were created along with a family design study, with cases matched to non-case full siblings. TBI was associated with higher risk of SCZ and BD (IRR=1.33 for SCZ, IRR=1.78 for BD). The association remained significant in the sibling comparison study. Moderate or severe TBI was associated with higher risk for both SCZ and BD compared to mild TBI. Older age at injury was associated with higher risk of SCZ and BD, and the effect of TBI was stronger in women than men. Findings indicate that TBI is a risk factor for both SCZ and BD with differential impact by age, severity and sex and that this association cannot be explained by familial confounding alone.

## 1. Introduction

### 1.1. Traumatic brain injury

Traumatic brain injury (TBI), defined as the alteration in brain function, or other evidence of brain pathology, caused by an external force, is a growing public health concern and has been described as a silent epidemic (Menon et al., 2010). It has an annual incidence of 1000 to 1300 cases per 100,000 people in Europe and North America (Dewan et al., 2018) and is responsible for over 8 million years of life lived with disability globally (James et al., 2019). TBI increases the risk for a wide range of adverse physical, neurological, cognitive, and social outcomes, both short and long term, including headache, fatigue, attention deficit, memory problems, dementia, Alzheimer’s disease, inferior school performance, unemployment, and poor social integration (Haarbauer-Krupa et al., 2021).

TBI is commonly classified into mild, moderate and severe subtypes (Hawryluk and Manley, 2015), based on either the Glasgow Coma Scale (GCS) score assigned in the first 24 h or the International Classification of Diseases (ICD) codes (Teasdale and Jennett, 1976). Studies have consistently shown mild TBI to be the most common subtype, constituting 80–90 % of all cases (Dewan et al., 2018; Maas et al., 2022a), and even mild TBI, previously considered to have little impact beyond the acute phase, has been shown to lead to long-term sequelae (Nelson et al., 2019; van der Vlegel et al., 2021).

Higher TBI risk is observed in males and people over age 75 or under four (Capizzi et al., 2020). The most common causes of TBI include falling, motor vehicle crashes, and struck-by or -against events, although their proportions vary considerably by demographic factors such as age and income settings (Faul et al., 2010; James et al., 2019; Maas et al., 2022b). For instance, for children four or under falling is the most common cause of TBI, whereas for people between 15 and 34 years old this is superseded by motor vehicle crashes (Faul et al., 2010).

### 1.2. Impact of TBI on risk of schizophrenia and bipolar disorder

TBI is associated with elevated risk for several psychiatric conditions, including depression, anxiety, post-traumatic stress disorder, and suicidality (Howlett et al., 2022). On the other hand, evidence linking TBI to the risk of schizophrenia (SCZ) and bipolar disorder (BD) has been scarce, inconsistent, and primarily based on small studies (Molloy et al., 2011; Orlovska et al., 2014; Yeh et al., 2020). A previous analysis with a restricted subsample of the Swedish Register born between 1973 and 1980 found no association between TBI and SCZ (Harrison et al., 2006); a 2017 meta-analysis also found no significant association between TBI and subsequent psychotic disorders, based on the five papers reviewed (Perry et al., 2016). On the other hand, the same meta-analysis found TBI to be associated with a higher risk of subsequent BD.

Additionally, studies have found little evidence for a dose-response relationship between TBI severity and risk for SCZ or BD (Molloy et al., 2011). Establishing causality remains difficult due to the potential confounding effects of genetic and environmental factors (Molloy et al., 2011). For example, people with a family history of SCZ were shown to have higher risk of TBI compared to those with a family history of BD (Malaspina et al., 2001). Also complicating the matter are the long prodromal phases of SCZ and BD: premorbid manifestations of SCZ and BD developing as early as childhood, such as lower IQ, attention difficulties, coordination problems, and unusual perceptual experiences, could result in higher risk of TBI (Poletti and Raballo, 2020; Trotta et al., 2015). Contradictory findings may also result from differential impact of TBI based on factors like sex, age at injury, and familial psychiatric history. For example, a positive association has been observed between age at first TBI and psychiatric hospitalisation (Molloy et al., 2011; Sariaslan et al., 2016). Finally, instead of a risk factor, some studies have suggested that TBI plays a risk-modifying role for individuals with higher genetic risk for SCZ (Kim, 2008).

### 1.3. Study aims

Capitalising on the large sample size, long-term follow-up, and comprehensive, high-quality data offered in the Swedish National Registers, this study aims to (1) examine the impact of TBI on the risk of subsequent SCZ and BD diagnoses incorporating both population-based methods and a sibling comparison design. Furthermore, we sought to determine (2) whether the association between TBI with SCZ and BD has a dose-response relationship by TBI severity, and (3) whether the association between TBI with SCZ and BD is moderated by sex, age at exposure, and familial history of SCZ and BD.

## 2. Methods

### 2.1. Study design and data sources

We identified all persons born in Sweden between 1973 and 1998 using the Total Population Register (TPR) to determine the study sampling frame. The National Patient Register (NPR) was used to identify all cases of SCZ and BD at least 15 years old at their first diagnosis. Individuals with both SCZ and BD diagnoses (*N* = 425) were included in both analyses.

*Nested case-control designs* for SCZ and BD were derived by matching each case (on birth year, sex, and birth county) to five individuals from the TPR without a SCZ or BD diagnosis, respectively, at the date of diagnosis of their matched case (index date); further information on the derivation of the matched case-control samples can be found in Robinson, et al. (2023). By matching each case to controls still at risk at their index date (incidence density sampling), we obtained a representative sample of person-time at risk for the disease permitting unbiased estimation of incidence rate ratios (IRR) (Pearce, 1993).

Family design using sibling comparison can account for unmeasured confounders from genetics and familial environment, which are assumed to be shared by siblings (D’Onofrio et al., 2013; Frisell, 2021; Sariaslan et al., 2016). *Sibling comparison designs* for SCZ and BD were created using the Multi-Generation Register (MGR) to identify biological full siblings of the SCZ and BD cases who were born during the study period and did not have a SCZ or BD diagnosis. Cases were excluded if either of their parents could not be identified or if they had no siblings born between 1973 and 1998. Non-case siblings were excluded if they had died before age 15. When a case had more than one non-case sibling, all non-case siblings were included. A flow chart showing inclusions and exclusions in the sibling comparison designs is shown in Supplementary Fig. 1.

For all analyses, we excluded individuals who were adopted or had a prior history of emigration from Sweden at the index date.

Exposure status was assessed by TBI diagnoses from the NPR. Family history of SCZ and BD was defined based on the MGR and NPR as a binary variable indicating participants with at least one 1st to 4th degree relative diagnosed with SCZ or BD, respectively. The Longitudinal Integrated Database for Health Insurance and Labour Market Studies (LISA) and the Labour statistics were used to derive highest attained parental education when a study participant was age 16 and average household disposable income level at ages 14–16. Date of birth was retrieved from the Medical Birth Register (MBR), emigration records from the Migration Register, and date of death from the Cause of Death Register.

### 2.2. Psychiatric outcomes

SCZ and BD diagnoses were based on ICD-8 (1973–1987), ICD-9 (1987–1996), or ICD-10 (1997–2013) (Supplementary Table 1). SCZ was defined as 295 in ICD-8 and 9 or F20 in ICD-10, excluding simple, latent types, acute schizophrenic episodes, and schizoaffective disorder. BD was defined as 296 in ICD-8 and 9 or F30, F31 in ICD-10, excluding unipolar depressive type, other, and unspecified types. Both inpatient and outpatient diagnoses were included, but outpatient records are only available since 2001.

### 2.3. Traumatic brain injury (TBI)

TBI was defined by ICD-8, −9, and −10 diagnostic codes using the guidelines proposed by the United States Centers for Disease Control and Prevention (CDC) (Supplementary Table 2) (Hedegaard et al., 2016). Mild TBI was defined as those only receiving a diagnosis of concussion (850 in ICD-8 and 9 or S06.0 in ICD-10), excluding those hospitalised for three days or more. Those who received any of the TBI-related ICD-10 codes other than that for concussion but did not undergo certain surgical procedures (insertion of pressure monitor, ventriculostomy, haematoma evacuation, or skull revision or ectomy) were classified as having moderate TBI, whereas those who underwent the aforementioned procedures were classed as having severe TBI (Carroll et al., 2012). As severe TBIs were rare, moderate and severe TBIs were combined for all statistical analyses.

In accordance with categories used by the US CDC (Faul et al., 2010), we stratified age at first TBI to 0–4, 5–14, and 15+, which reflects differences in injury risk, injury mechanism, and neuroplasticity. For the statistical analysis, we dichotomised age into 0–14 and 15 and above, as TBI before age 5 was too rare to be analysed separately.

### 2.4. Statistical analysis

For the nested case-control design, conditional logistic regression was performed to obtain odds ratios for SCZ and BD, which are interpreted as incidence rate ratios (IRR). We estimated the IRR for SCZ and BD for TBI of any type, TBI of different severity, and at different ages, all compared to no TBI. We also estimated the IRR for TBI of different severity and at different ages, using an exposure that cross-tabulated TBI severity and age at TBI to create four categories. We report both crude IRR estimates and that adjusted for parental income level and parental highest education level. Following Robinson, et al. (2024), parental income level was stratified into quartiles and parental highest education level has four categories (compulsory, upper secondary, higher education, and postgraduate) (Robinson et al., 2024).

For the sibling comparison sample, we fitted stratified Cox proportional hazard models stratified by family (sibships), with attained age as underlying time scale, to obtain hazard ratios (HR) of any TBI, mild TBI, moderate or severe TBI, TBI sustained younger than 15 years old, and TBI sustained at 15 years old or older. We did not fit a Cox model for the cross-tabulated exposure because of insufficient power.

We added interaction terms to the models to obtain separate effect estimates for men and women and for participants with and without familial psychiatric history.

For all analyses, we defined statistical significance as a p-value below 0.05.

### 2.5. Sensitivity analyses

We used non-TBI falls as a negative control exposure to examine whether the association of TBI with SCZ and BD may be confounded by shared genetic predisposition or environmental risk factors between general injury and SCZ or BD, or by prodromal manifestations of SCZ or BD traits rendering individuals more prone to injuries. Non-TBI-related falls were defined using ICD codes (880–888 in ICD-8 or 9 or W00-W19 in ICD-10) and excluding TBI in the same episode. Conditional logistic regression models were fitted in the nested case-control for non-TBI-related falls as previously described.

Furthermore, to investigate the potential impact of reverse causality, we tested the associations between having had a SCZ and BD diagnosis and subsequent risk for TBI. By treating the nested case-control designs as a matched cohort designs, we modelled subsequent TBI as a time-to-event outcome, with time since SCZ/BD diagnosis as the time scale. Using Cox proportional hazard models stratified by matched sets, we estimated HR of any TBI.

Data management was conducted in SAS (version 9.4) and Stata/BE (version 17.0) (SAS Institute, 1985; StataCorp, 2021). Statistical analyses were performed in R (version 4.0.4) using the integrated development environment RStudio, with the survival package (version 3.5–5) (R Core Team, 2021; Therneau and Grambsch, 2000).

The data linkage from the Swedish national registers was previously approved by the Regional Ethics Review Board, Stockholm, Sweden (DNR 2013/862–31/5). No additional ethical approval was required for this study, and no informed consent was required for the analysis of anonymised register data.

## 3. Results

### 3.1. Descriptive statistics

We identified 4184 SCZ cases and 18,681 BD cases, which were matched to 20,920 and 93,405 controls, respectively (Table 1). The sibling comparison sample for SCZ had 2812 cases and 4373 non-case siblings; that for BD had 13,337 cases and 19,726 non-case siblings; the ratio between non-case siblings and cases was 1.56 for SCZ and 1.48 for BD. We found more men in the SCZ samples (66 % in the case-control sample and 57 % in the sibling samples) and more women in the BD samples (66 % in the case-control sample and 56 % in the sibling samples).

Across all samples, the proportion of individuals who had experienced TBI was consistently higher among SCZ and BD cases compared to their controls (Table 1). Mild TBI constituted the majority of all TBI cases (85–90 %), whereas the proportion of severe TBI was below 2 % in all samples, regardless of case-control status. In the BD case-control sample, TBI sustained at 15 years or older was more likely to be moderate or severe (11.99 %) than TBI sustained before age 15 (9.51 %). There was no such difference in the SCZ case-control sample.

### 3.2. Association between TBI and risk of SCZ and BD

**Fig. 1.**
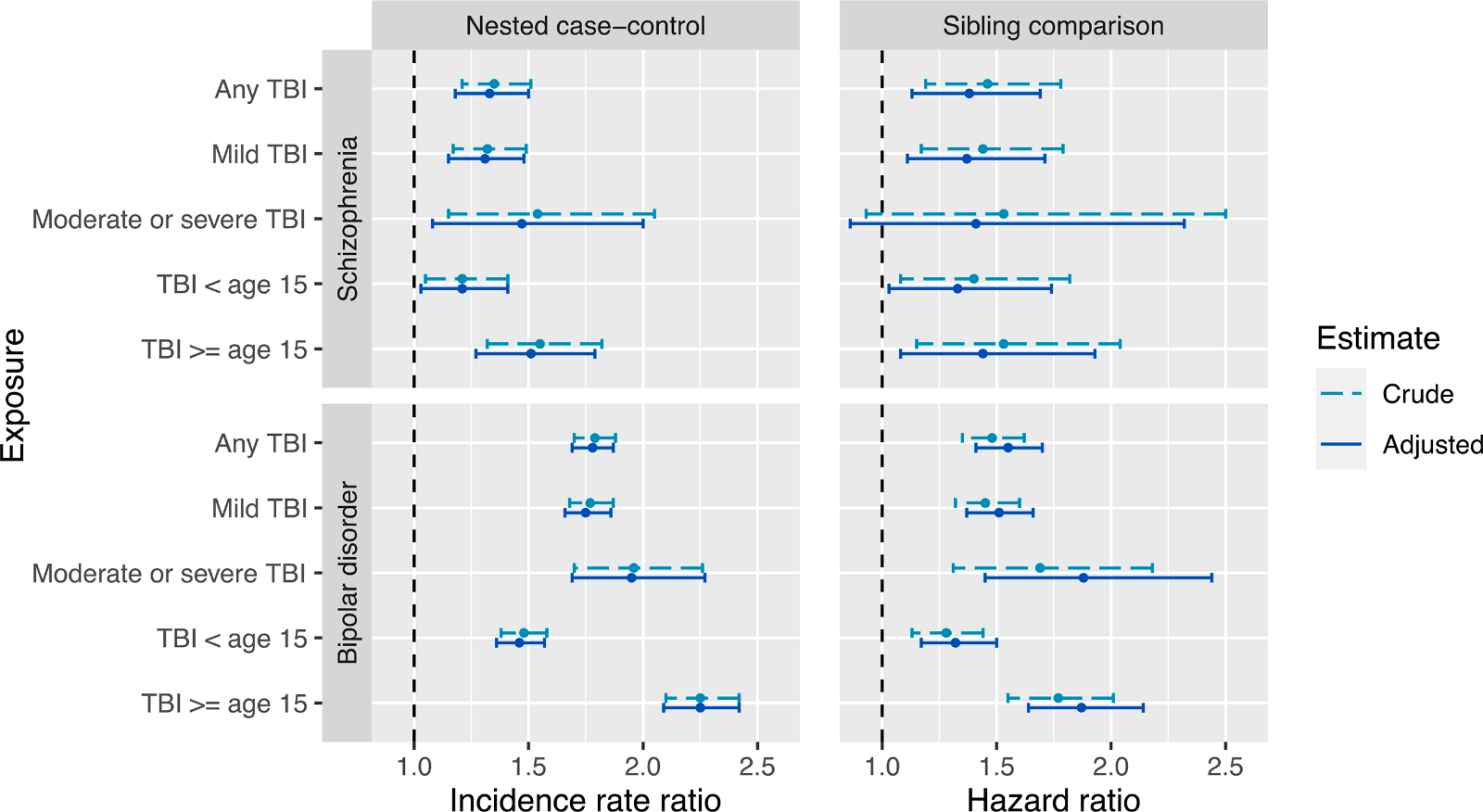
Effect estimates of TBI from the nested case-control study (interpreted as incidence rate ratio (IRR)) and sibling comparison study (interpreted as hazard ratios (HR)).

In the nested case-control samples, experiencing any TBI was statistically significantly associated with a higher risk of SCZ (adjusted IRR 1.33, p-value < 0.001) and BD (aIRR 1.78, *p* < 0.001) (Fig. 1 and Supplementary Table 3).

Compared to mild TBI, moderate or severe TBI was associated with higher relative risk for both SCZ (aIRR 1.31, *p* <0.001 for mild TBI; aIRR 1.47, *p* = 0.013 for moderate and severe TBI) and BD (aIRR 1.75, *p* < 0.001 for mild TBI; aIRR 1.95, *p* < 0.001 for moderate and severe TBI). Later age at first TBI (≥15 years) was also associated with a higher relative risk for both SCZ (aIRR 1.21, *p* = 0.019 for early TBI; aIRR 1.51, *p* < 0.001 for later TBI) and BD (aIRR 1.46, *p* < 0.001 for early TBI; aIRR 2.25, *p* < 0.001 for later TBI), compared to TBI sustained before age 15. Using the cross-tabulated exposure of TBI severity and age at TBI, the risk estimate for later-age TBI was found to be larger than that for early-age TBI regardless of TBI severity, and moderate and severe TBI had a larger effect than mild TBI regardless of age of TBI (Supplementary Table 3). Adjustments for parental income and parental highest education resulted in no appreciable changes to the effect estimates.

Using sibling comparison samples to adjust more comprehensively for familial confounding, we reported HR to complement the above results (Fig. 1 and Supplementary Table 3). TBI remained significantly associated with both SCZ (aHR=1.38, *p* = 0.002) and BD (aHR=1.55, *p* < 0.001); moderate or severe TBI was again found to be associated with higher SCZ and BD risks, compared to mild TBI. TBI sustained at age 15 or older was associated with higher relative risks for both disorders, albeit the relative excess risk was attenuated compared to the nested case-control study.

### 3.3. Differences by sex and family history

We found the association between TBI and BD to be statistically significantly stronger in women than in men (Table 2). This was true for any TBI exposure (aIRR 1.88, *p* < 0.001 for women, aIRR 1.63, *p* < 0.001 for men; interaction *p* = 0.0041) and for TBI sustained at different ages (interaction *p* = 0.0021, Table 3). While the risk estimate for mild TBI was also larger in women, that for moderate and severe TBI was similar between the two sexes. In the case of SCZ, while the moderating effect of sex was not statistically significant (*p* = 0.26), the risk of SCZ associated with TBI was 15 % higher in women than in men, similar to the 16 % relative excess risk between women and men for BD.

In contrast, we did not find evidence that a family history of SCZ or BD moderated the risk of psychiatric diagnosis subsequent to TBI (all interaction *p* > 0.05; Table 3). A larger IRR was estimated for the association between TBI and SCZ for people with at least one relative diagnosed with SCZ (crude IRR 1.52, *p* = 0.031) than for people with no affected relatives (cIRR 1.35, *p* < 0.001). For BD, the IRR estimate was marginally smaller for those with an affected relative, compared to those with no affected relatives.

### 3.4. Sensitivity analyses

As a negative control exposure, non-TBI falls were not associated with a higher risk of subsequent SCZ (aIRR 0.91, *p* = 0.058, Supplementary Table 4), but were associated with a statistically significantly higher risk for subsequent BD (aIRR 1.26, *p* < 0.001). However, the effect size for non-TBI falls was distinctly smaller than for TBI and BD (aIRR 1.79; Table 2).

Reversing the effect direction, we found that diagnoses of SCZ or BD were associated with a statistically significantly increased risk of subsequent TBI incidents (SCZ aHR=1.40, *p* = 0.005; BD aHR=2.18, *p* < 0.001 for BD; Supplementary Table 5).

## 4. Discussion

Utilising data from nationwide population-based registers, we examined the association between TBI and subsequent risk of SCZ and BD. We included 4184 SCZ and 18,681 BD cases during a total follow-up time of 40 years and compared them to two sets of control groups: unrelated matched controls and their non-case siblings. This is, to our knowledge, the largest study with the longest follow-up time on this topic to date.

### 4.1. Association between TBI and SCZ/BD

There were five main findings from this study. First, we found that a history of TBI was associated with a higher risk of both SCZ and BD: TBI accounted for a 33 % increase in the risk of SCZ and a 78 % increase in the risk of BD. While our findings suggested a small overall effect from TBI, this was comparable to population-based estimates reported for other environmental risk factors for SCZ and BD and reflects the complex pathogenesis of these disorders (Robinson et al., 2023).

The validity of the findings from the case-control study was strengthened by the congruent results from the sibling comparison study, which accounted for genetic and familial confounding more comprehensively than adjustment for individual factors. Comparing estimates from full siblings with those from the nested case-control study, the effect size for any TBI was similar for SCZ (HR=1.38 compared to IRR=1.33) and attenuated for BD (HR=1.55 compared to IRR=1.78) while remaining statistically significant. The diminished but significant HR for BD was consistent with a causal effect of TBI on psychiatric risk that could not be completely explained by shared genetic and environmental factors between siblings.

### 4.2. Dose-response relationship

Second, we found evidence that moderate or severe TBI was associated with higher risk of SCZ and BD than mild TBI. While no dose-response effect has previously been reported for TBI severity and SCZ or BD, there have been similar findings for overall risk for psychiatric disorders and depression (Kerr et al., 2012; Max et al., 2021). A dose-response relationship is an important element in establishing a causal relationship, as it yields evidence of potential biological plausibility and gradient. It is possible that the stronger effect from more severe TBI compared to mild TBI reflected a larger degree of neurological damage.

Neurobiological studies have shed light on possible mechanisms through which TBI impacts the risk of SCZ and BD. For example, TBI has been shown to increase blood-brain barrier permeability to inflammatory molecules, which have in turn been shown to contribute to the aetiology and clinical course of SCZ and BD (Pollak et al., 2018; Zhao et al., 2022). Axonal degeneration, one of the most common pathological features in TBI that may continue years after injury (Johnson et al., 2013), has also been suggested to play a role in the pathophysiology of SCZ and BD (Kelly et al., 2017; Zalesky et al., 2011). However, much remains to be done in terms of establishing a clear pathophysiological mechanism, and our findings underscore the need for further research on this topic.

### 4.3. Subgroup differences

Third, we found that the effect of TBI sustained at ≥15 years old was stronger than that of TBI sustained earlier. One possible explanation is that this is due to the more severe TBI sustained at older age, which was true for the BD case-control sample but not for the SCZ sample. It may also be the result of greater neuroplasticity in early life, allowing recovery to a larger extent after injury (Su et al., 2016). While previous studies have reported a wide range of onset latency of psychosis after TBI (Batty et al., 2013), it is possible that TBI sustained at the critical age of developing SCZ and BD incurs a greater risk.

Fourth, the effect of TBI on the risk for BD was found to be larger in women than in men. A similar pattern of excess risk for women was also found for SCZ albeit non-significant, potentially due to lower statistical power. This sex difference is in agreement with other studies which found that, following TBI, women have poorer outcomes in terms of cognitive function and mental health (Gupte et al., 2019). Possible reasons for this include differences in the stress response (hypothalamic-pituitary-adrenal) axis, which has been shown to produce stronger inflammatory response in female versus male rodents, and the stronger impact possessing the APOE4 allele has on post-TBI outcome for women than for men (Ponsford et al., 2011; Späni et al., 2018).

Finally, we found no moderating effect from familial psychiatric history, indicating that the psychiatric risk induced by TBI did not differ between people with or without relatives affected by the same disorder. This suggested that the observed association between TBI with SCZ and BD could not be interpreted as merely the translation of inherited psychiatric risk.

### 4.4. Confounding and reverse causality

Our finding that non-TBI falls, a negative control assumed to have minimal neuropsychiatric impact, was associated with a higher risk of BD (but not SCZ) suggested that the association between TBI and BD may be partly confounded by prodromal symptoms manifesting before psychiatric diagnosis, such as attention difficulties and impulsivity, which underlie both risk of injury and risk of BD (Trotta et al., 2015). Nevertheless, the effect estimates for non-TBI falls were considerably smaller than those of TBI, suggesting that such confounding effects did not fully account for the observed psychiatric risk associated with TBI.

SCZ and BD diagnoses were also found to be associated with higher risk of subsequent TBI, with a notably large effect for BD. This is not unexpected, as the higher risks of violence and self-harm among people with SCZ and BD have been shown to lead to increased injury risk (Nielssen et al., 2012). However, this once again highlighted the possibility of confounding effects from shared risk factors of TBI and SCZ or BD. Furthermore, it pointed toward the potential impact of reverse causality, in the case that the association between TBI with SCZ and BD was partly due to the increased risk of TBI caused by undiagnosed psychiatric disorders. Nevertheless, our finding that TBI before age 15, the age of earliest psychiatric diagnosis defined in our study, was associated with higher risk of psychiatric diagnoses argues against this.

### 4.5. Strengths and limitations

By using data from the Swedish national registers, this study has good statistical power due to its large sample size and long follow-up. Furthermore, its coverage reduces selection bias and increases generalisability. Diagnoses in these registers have been shown to have good validity (Ludvigsson et al., 2011). Finally, the sibling comparison design and negative control exposure provided strong validation for our findings by accounting for important unmeasured confounders.

There were also several limitations. Primary care diagnoses were not included in our data, and outpatient diagnoses were only recorded from 2001. This may have led to missing milder TBI exposures and some outcome diagnoses. In terms of measurement, a variety of definitions have been used for TBI cases in previous studies. While the gold standard for TBI triage is the Glasgow Coma Scale (GCS) score (Teasdale and Jennett, 1976), we adopted the US CDC definition of severities based on ICD codes and operation records, to which we have access. This nevertheless calls for caution when comparing our findings with other studies.

Furthermore, although the family design allowed for better adjustment for familial confounders, it is not comprehensive, as e.g. siblings who were born at different times may not have grown up in the same environments, as is assumed. Researchers have also warned about additional biases induced by family design, such as cross-sibling interactions, where the elder sibling affects the propensity to exposure or outcome of the younger sibling (Frisell, 2021; Sjölander et al., 2022). The direction and magnitude of these biases are difficult to estimate. In our case, for example, TBI in the firstborn might lead to the parents taking more preventive measures to avoid similar injuries in the future. Alternatively, the caring burden for the firstborn’s TBI could result in negligence of the younger siblings. Therefore, it is important to note that estimates from sibling comparison studies are not bias-free. Instead, it is the triangulation of findings from familial and conventional study designs that provided mutually validating evidence.

Finally, despite our best efforts, it was not possible to completely rule out the potential biases of residual confounding effects and especially reverse causality. While this would not invalidate the clinical significance of our finding that patients with a history of TBI should receive close attention for psychiatric symptoms and potential subsequent psychiatric diagnoses, the establishment of a causal relationship will require further research efforts.

## 5. Conclusions

This study finds evidence for an association between TBI and subsequent risk for SCZ and BD. Using a family design study, which adjusted for unmeasured confounding effects of genetic factors and shared living environment, we further strengthened the evidence for this association. We also found evidence suggesting a dose-response relation between TBI severity and the risk for these disorders, and a positive association between age at TBI and subsequent risk of SCZ and BD. Additionally, the risk associated with TBI was stronger in women than men. The associations between TBI and subsequent SCZ and BD should prompt clinicians to monitor clinical course and potential psychiatric symptoms in people with a history of TBI, particularly the vulnerable groups identified in our study.

## Supplementary Material

Supplement

## Figures and Tables

**Table 1:** Descriptive statistics for the study samples.

	Schizophrenia			Bipolar disorder		
	Matched case control sample					

	All *N* = 25,104	Case *N* = 4184	Control *N* = 20,920	All *N* = 112,086	Case *N* = 18,681	Control *N* = 93,405

Any TBI	2080 (8.29 %)	432 (10.33 %)	1648 (7.88 %)	9348 (8.34 %)	2356 (12.60 %)	6992 (7.49 %)
Severity (%)						
Mild	1814 (87.21 %)	371 (85.88 %)	1443 (87.56 %)	8356 (89.42 %)	2086 (88.65 %)	6270 (89.67 %)
Moderate	251 (12.07 %)	55 (12.73 %)	196 (11.89 %)	948 (10.14 %)	258 (10.96 %)	690 (9.87 %)
Severe	15 (0.72 %)	6 (1.39 %)	9 (0.55 %)	41 (0.44 %)	9 (0.38 %)	32 (0.46 %)
Age at injury						
Mean	13.30	14.23	13.06	13.52	15.10	12.99
<5 (%)	349 (16.78 %)	66 (15.28 %)	283 (17.17 %)	1657 (17.73 %)	319 (13.56 %)	1338 (19.14 %)
5–14 (%)	857 (41.20 %)	164 (37.96 %)	693 (42.05 %)	3652 (39.08 %)	836 (35.53 %)	2816 (40.27 %)
≥15 (%)	874 (42.02 %)	202 (46.76 %)	672 (40.78 %)	4036 (43.19 %)	1198 (50.91 %)	2838 (40.59 %)

**Full sibling comparison sample**						

	All *N* = 7185	Case *N* = 2812	Control *N* = 4373	All *N* = 33,063	Case *N* = 13,337	Control *N* = 19,726

Any TBI	671 (9.34 %)	285 (10.14 %)	386 (8.83 %)	3674 (11.11 %)	1662 (12.46 %)	2012 (10.20 %)
Severity (%)						
Mild	582 (86.74 %)	243 (85.26 %)	339 (87.82 %)	3288 (89.49 %)	1484 (89.29 %)	1804 (89.66 %)
Moderate	82 (12.22 %)	37 (12.98 %)	45 (11.66 %)	371 (10.10 %)	171 (10.29 %)	200 (9.94 %)
Severe	7 (1.04 %)	5 (1.75 %)	2(0.52 %)	15 (0.41 %)	7 (0.42 %)	8 (0.40 %)
Age at injury						
Mean	14.88	13.85	15.64	14.62	15.07	14.25
<5 (%)	99 (14.75 %)	45 (15.79 %)	54 (13.99 %)	552 (15.02 %)	222 (13.36 %)	330 (16.40 %)
5–14 (%)	236 (35.17 %)	107 (37.54 %)	129 (33.42 %)	1322 (35.98 %)	593 (35.68 %)	729 (55.14 %)
≥15 (%)	336 (50.07 %)	133 (46.67 %)	203 (52.59 %)	1800 (48.99 %)	847 (50.97 %)	953 (47.37 %)

**Table 2:** Risk of SCZ and BD by TBI, injury severity, and age of injury, as estimated separately for men and women.

	Women						Men						Wald P^1^
	Unadjusted			Adjusted ^2^			Unadjusted			Adjusted ^2^			
	IRR	95 % CI		IRR	95 % CI		IRR	95 % CI		IRR	95 % CI		

**Schizophrenia**													
Any TBI	1.50	(1.18, 1.81)	<0.001	1.44	(1.16, 1.80)	<0.001	1.30	(1.13, 1.47)	<0.001	1.29	(1.12, 1.48)	<0.001	0.26
TBI by severity													0.50
Mild	1.44	(1.15, 1.80)	0.001	1.39	(1.10, 1.76)	0.007	1.28	(1.11, 1.47)	0.001	1.28	(1.10, 1.48)	0.001	
Moderate or Severe	2.01	(1.12, 3.59)	0.019	1.96	(1.06, 3.61)	0.031	1.41	(1.01, 1.97)	0.042	1.35	(0.95, 1.92)	0.100	
Age at first TBI													0.46
<15	1.36	(1.04, 1.78)	0.027	1.35	(1.01, 1.79)	0.041	1.16	(0.97, 1.38)	0.108	1.15	(0.96, 1.39)	0.136	
≥15	1.74	(1.26, 2.41)	0.001	1.60	(1.14, 2.25)	0.006	1.49	(1.23, 1.79)	<0.001	1.47	(1.21, 1.79)	<0.001	
**Bipolar disorder**													
Any TBI	1.90	(1.78, 2.03)	<0.001	1.88	(1.76, 2.01)	<0.001	1.64	(1.51, 1.77)	<0.001	1.63	(1.50, 1.77)	<0.001	0.0041
TBI by severity													0.0040
Mild	1.90	(1.78, 2.03)	<0.001	1.87	(1.75, 2.01)	<0.001	1.59	(1.46, 1.73)	<0.001	1.59	(1.45, 1.73)	<0.001	
Moderate or Severe	1.91	(1.56, 2.34)	<0.001	1.94	(1.57, 2.39)	<0.001	2.01	(1.65, 2.45)	<0.001	1.96	(1.59, 2.42)	<0.001	
Age at first TBI													0.0021
<15	1.54	(1.41, 1.68)	<0.001	1.50	(1.37, 1.64)	<0.001	1.38	(1.24, 1.54)	<0.001	1.39	(1.24, 1.56)	<0.001	
≥15	2.49	(2.27, 2.73)	<0.001	2.51	(2.28, 2.76)	<0.001	1.97	(1.76, 2.19)	<0.001	1.94	(1.73, 2.18)	<0.001	

1 P-value for Wald test for the significance of the interaction term.

2 Adjusted for parental education and parental income.

**Table 3:** Risk of SCZ and BD by TBI, injury severity, and age of injury, as estimated separately for people with and without affected relatives.

	With an affected relative			Without affected relatives			Wald P^1^
	IRR	95 % CI	P	IRR	95 % CI	P	

**Schizophrenia**							
Any TBI	1.35	(1.17, 1.56)	<0.001	1.52	(1.04, 2.23)	0.031	0.56
TBI by severity							0.84
Mild	1.32	(1.13, 1.54)	<0.001	1.49	(0.99, 2.25)	0.053	
Moderate or Severe	1.56	(1.07, 2.27)	0.020	1.76	(0.65, 4.84)	0.271	
Age at first TBI							0.46
<15	1.26	(1.04, 1.52)	0.018	1.17	(0.70, 1.94)	0.551	
≥15	1.48	(1.20, 1.83)	<0.001	2.14	(1.21, 3.77)	0.009	
**Bipolar disorder**							
Any TBI	1.82	(1.70, 1.95)	<0.001	1.79	(1.59, 2.01)	<0.001	0.78
TBI by severity							0.72
Mild	1.80	(1.68, 1.93)	<0.001	1.74	(1.54, 1.97)	<0.001	
Moderate or Severe	1.98	(1.63, 2.40)	<0.001	2.23	(1.60, 3.13)	<0.001	
Age at first TBI							0.90
<15	1.51	(1.38, 1.66)	<0.001	1.53	(1.32, 1.79)	<0.001	
≥15	2.27	(2.06, 2.49)	<0.001	2.17	(1.83, 2.57)	<0.001	

1 P-value for Wald test for the significance of the interaction term.
