# Supplementary material for: Impact of traumatic brain injury on risk for schizophrenia and bipolar disorder
Source: Psychiatry Res. Author manuscript; Available in PMC 2024 Sep 1. (PMC11321911; doi:10.1016/j.psychres.2024.115990)
Supplement: Supplement [file NIHMS2009746-supplement-Supplement.docx]

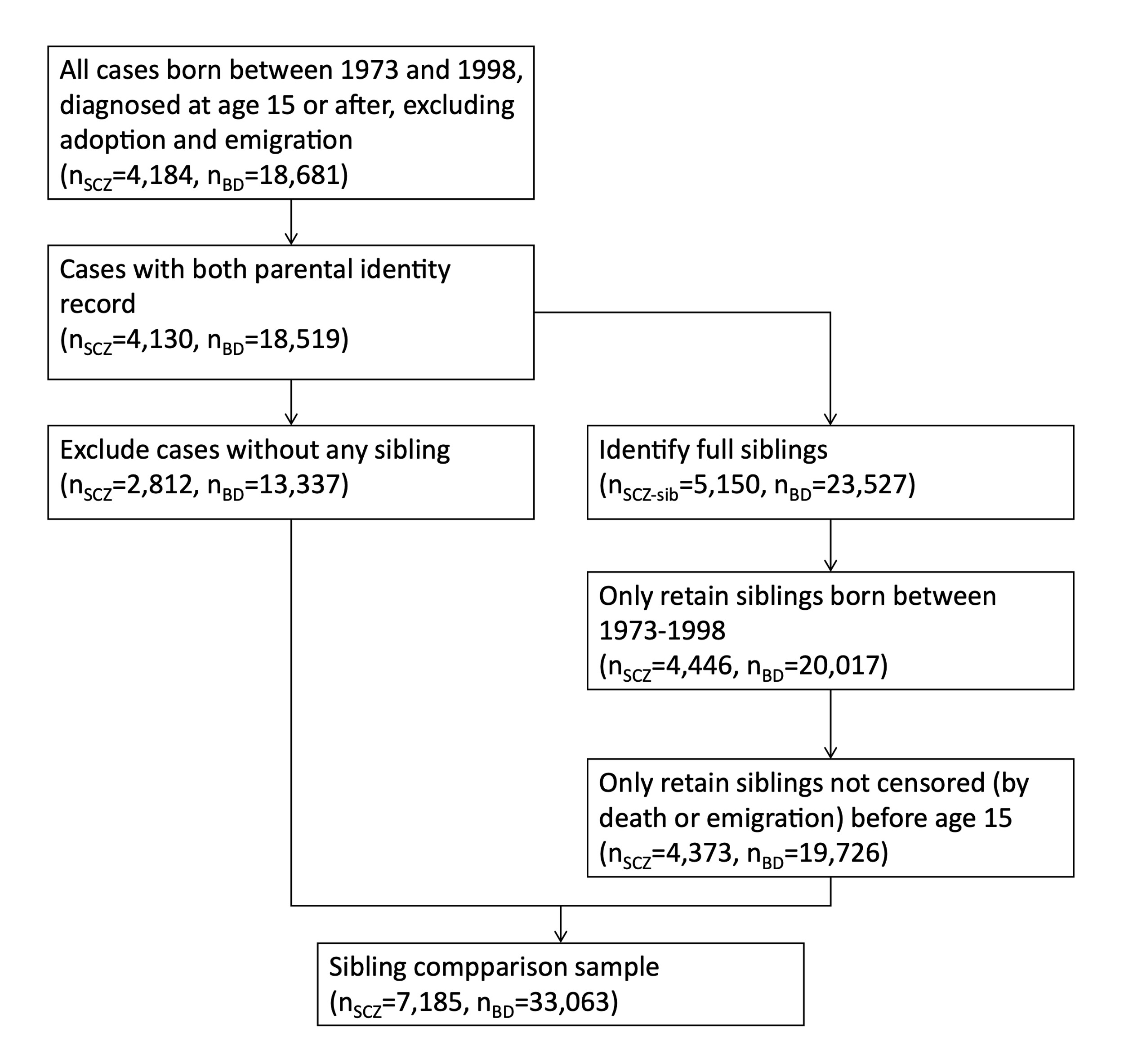


Supplementary Figure 1 Flow chart for the identification of the sibling comparison samples.

Supplementary Table 1 Diagnostic codes for schizophrenia and bipolar disorder.

| Variable | ICD-8 | ICD-9 | ICD-10 |
| --- | --- | --- | --- |
| Schizophrenia | 295 (excluding .0, .4, .5, .7) | 295 2 | F20  (excluding .4, .6) |
| Bipolar disorder | 296  (excluding .2, .8, .9) | 296  (excluding .1, .8, .9) | F30, F31 |

Supplementary Table 2 Diagnostic codes for traumatic brain injury

| Variable | ICD-8 | ICD-9 | ICD-10 ^1^ |
| --- | --- | --- | --- |
| Any TBI | 800, 801, 803, 804, 850, 851, 852, 853, 854 | 800, 801, 803, 804, 850, 851, 852, 853, 854 | S02.0-, S02.1-, S02.7-, S02.8-, S02.9-, S04.0, S06–, S07.1 |
| Mild TBI (concussion) | 850 only  (excluding hospitalisation for 3 days or more) | 850 only  (excluding hospitalisation for 3 days or more) | S06.0 only  (excluding hospitalisation for 3 days or more) |
| Moderate TBI | 800, 801, 803, 804, 850, 851, 852, 853, 854  (excluding hospitalisation less than 3 days; excluding those with OP codes defined below) | 800, 801, 803, 804, 850, 851, 852, 853, 854  (excluding hospitalisation less than 3 days; excluding those with KVÅ codes defined below) | S02.0-, S02.1-, S02.7-, S02.8-, S02.9-, S04.0, S06–, S07.1  (excluding hospitalisation less than 3 days; excluding those with KVÅ codes defined below) |
| Severe TBI | 800, 801, 803, 804, 850, 851, 852, 853, 854  **and**  OP SV 1964-1996 codes ^2^:  0100, 0101, 0109, 0130, 0227, 0228 | 800, 801, 803, 804, 850, 851, 852, 853, 854  **and**  KVÅ codes ICD-9 2:  0124, 0125, 0118 | S02.0-, S02.1-, S02.7-, S02.8-, S02.9-, S04.0, S06–, S07.1  **and**  KVÅ codes ^2^:  AAA20, AAA25, AAA27, AAF00, AAD00, AAD05, AAD15, AAD30, AAD40, AAD99, AAK80 |
| ^1^ ICD-10 codes followed by hyphen (-) indicates any fourth, fifth, or sixth character.  ^2^ These codes are or were unique to the Swedish National Registers that denotes specific surgical procedures: 0100 test drilling; 0101 exploratory craniotomy; 0109 other operations related to craniotomy; 0130 craniotomy + evacuation or drainage; 0227 intracranial pressure monitoring (intraventricular); 0228 other operations related to intracranial pressure; 0124 test drilling, exploratory craniotomy, or craniotomy + evacuation or drainage; 0125 other operations related to craniotomy; 0118 intracranial pressure monitoring (intraventricular or other); AAA20 Insertion of intracranial pressure gauge; AAA25 Insertion of epidural pressure gauge; AAA27 Insertion of intracerebral pressure monitor; AAF00 Ventriculostomy; AAD00 Evacuation of epidural hematoma; AAD05 Evacuation of acute subdural hematoma; AAD15 Evacuation of traumatic intracerebral hematoma; AAD30 Revision of penetrating or perforating skull injury; AAD40 Revision of skull fracture; AAD99 Revision of skull fracture or other surgery due to cranial or intracranial traumatic change; AAK80 Partial ectomy of the skull roof. | | | |

Supplementary Table 3 Conditional logistic regression for the risk of SCZ and BD by TBI, injury severity, and age of injury, including using the combined exposure variable between TBI severity and age of frst TBI in the nested case-control study

| **Schizophrenia** | | | | | | |
| --- | --- | --- | --- | --- | --- | --- |
|  | Unadjusted | | | Adjusted | | |
| *Incidence rate ratio (IRR) from nested case-control study* | | | | | | |
| Comparison: no TBI | IRR | 95% CI | P | IRR | 95% CI | P |
| Any TBI | 1.35 | (1.21, 1.51) | <0.001 | 1.33 | (1.18, 1.50) | <0.001 |
| TBI by severity |  |  |  |  |  |  |
| Mild | 1.32 | (1.17, 1.49) | <0.001 | 1.31 | (1.15, 1.48) | <0.001 |
| Moderate/severe | 1.54 | (1.15, 2.05) | 0.004 | 1.47 | (1.08, 2.00) | 0.013 |
| Age of first TBI |  |  |  |  |  |  |
| <15 | 1.21 | (1.05, 1.41) | 0.011 | 1.21 | (1.03, 1.41) | 0.019 |
| ≥15 | 1.55 | (1.32, 1.82) | <0.001 | 1.51 | (1.27, 1.79) | <0.001 |
| Combined |  |  |  |  |  |  |
| <15, mild | 1.19 | (1.02, 1.40) | 0.029 | 1.20 | (1.02, 1.42) | 0.032 |
| <15, moderate/severe | 1.35 | (0.91, 2.00) | 0.135 | 1.25 | (0.82, 1.91) | 0.306 |
| ≥15, mild | 1.51 | (1.27, 1.80) | <0.001 | 1.47 | (1.22, 1.76) | <0.001 |
| ≥15, moderate/severe | 1.81 | (1.18, 2.76) | 0.006 | 1.79 | (1.15, 2.79) | 0.009 |
| *Hazard ratio (HR) from sibling comparison study* | | | | | | |
| Any TBI | 1.46 | (1.19, 1.78) | <0.001 | 1.38 | (1.13, 1.69) | 0.002 |
| TBI by severity |  |  |  |  |  |  |
| Mild | 1.44 | (1.17, 1.79) | <0.001 | 1.37 | (1.11, 1.71) | 0.004 |
| Moderate/severe | 1.53 | (0.93, 2.50) | 0.093 | 1.41 | (0.86, 2.32) | 0.177 |
| Age of first TBI |  |  |  |  |  |  |
| <15 | 1.40 | (1.08, 1.82) | 0.010 | 1.33 | (1.03, 1.74) | 0.030 |
| ≥15 | 1.53 | (1.15, 2.04) | 0.004 | 1.44 | (1.08, 1.93) | 0.014 |
| **Bipolar disorder** | | | | | | |
|  | Unadjusted | | | Adjusted | | |
| *Incidence rate ratio (IRR) from nested case-control study* | | | | | | |
| Comparison: no TBI | IRR | 95% CI | P | IRR | 95% CI | P |
| Any TBI | 1.79 | (1.70, 1.88) | <0.001 | 1.78 | (1.69, 1.87) | <0.001 |
| TBI by severity |  |  |  |  |  |  |
| Mild | 1.77 | (1.68, 1.87) | <0.001 | 1.75 | (1.66, 1.86) | <0.001 |
| Moderate/severe | 1.96 | (1.70, 2.26) | <0.001 | 1.95 | (1.69, 2.27) | <0.001 |
| Age of first TBI |  |  |  |  |  |  |
| <15 | 1.48 | (1.38, 1.58) | <0.001 | 1.46 | (1.36, 1.57) | <0.001 |
| ≥15 | 2.25 | (2.10, 2.42) | <0.001 | 2.25 | (2.09, 2.42) | <0.001 |
| Combined |  |  |  |  |  |  |
| <15, mild | 1.46 | (1.36, 1.57) | <0.001 | 1.44 | (1.34, 1.56) | <0.001 |
| <15, moderate/severe | 1.64 | (1.33, 2.01) | <0.001 | 1.60 | (1.30, 1.98) | <0.001 |
| ≥15, mild | 2.24 | (2.08, 2.41) | <0.001 | 2.23 | (2.07, 2.42) | <0.001 |
| ≥15, moderate/severe | 2.34 | (1.92, 2.84) | <0.001 | 2.38 | (1.94,2.92) | <0.001 |
| *Hazard ratio (HR) from sibling comparison study* | | | | | | |
| Any TBI | 1.48 | (1.35, 1.62) | <0.001 | 1.55 | (1.41, 1.70) | <0.001 |
| TBI by severity |  |  |  |  |  |  |
| Mild | 1.45 | (1.32, 1.60) | <0.001 | 1.51 | (1.37, 1.66) | <0.001 |
| Moderate/severe | 1.69 | (1.31, 2.18) | <0.001 | 1.88 | (1.45, 2.44) | <0.001 |
| Age of first TBI |  |  |  |  |  |  |
| <15 | 1.28 | (1.13, 1.44) | <0.001 | 1.32 | (1.17, 1.50) | <0.001 |
| ≥15 | 1.77 | (1.55, 2.01) | <0.001 | 1.87 | (1.64, 2.14) | <0.001 |
| ^1^ For nested case-control study: adjusted for parental income level and parental education level in the model, adjusted for sex, birth year, birthplace by matching; for sibling comparison study: adjusted for sex. | | | | | | |

Supplementary Table 4 The association between non-TBI related falling and risk of schizophrenia and bipolar disorder from conditional logistic regression.

|  | Schizophrenia | | | Bipolar disorder | | |
| --- | --- | --- | --- | --- | --- | --- |
|  | IRR | 95% CI | P | IRR | 95% CI | P |
| Non-TBI related falling | 0.91 | (0.83, 1.00) | 0.058 | 1.26 | (1.21, 1.32) | <0.001 |

Supplementary Table 5 The association between schizophrenia and bipolar disorder diagnoses and subsequent TBI from stratified Cox proportional hazard model.

|  | Post-psychiatric disorder TBI | |  |
| --- | --- | --- | --- |
|  | HR | 95% CI | P |
| Schizophrenia | 1.40 | (1.10, 1.77) | 0.005 |
| Bipolar disorder | 2.18 | (1.92, 2.47) | <0.001 |
